# Successful Treatment of Two Cases of Refractory Macular Holes With Intentional Retinal Detachment and Posterior Capsule Transplantation

**DOI:** 10.7759/cureus.93808

**Published:** 2025-10-04

**Authors:** Miki Sato, Hiroyuki Nakashizuka, Chiho Shoda, Ryusaburo Mori

**Affiliations:** 1 Ophthalmology, Nihon University Hospital, Tokyo, JPN; 2 Ophthalmology, Nihon University School of Medicine, Tokyo, JPN

**Keywords:** intentional retinal detachment, posterior capsule transplantation, posterior lens capsular flap transplantation, refractory macular hole, subretinal fluid application

## Abstract

We present two cases of refractory macular holes that were successfully treated with intentional retinal detachment and posterior lens capsule transplantation. Case 1 involves a 60-year-old man with a three-year history of vision loss, who was diagnosed with macular holes in both eyes. Initial treatments, including vitrectomy and internal limiting membrane translocation in the right eye, led to hole closure, but the left eye required reoperation. In the second surgery, the left eye underwent intentional retinal detachment combined with posterior capsule transplantation and tamponade with 9% octafluoropropane. This resulted in the successful closure of the macular hole and improved visual acuity. Case 2 involves a 69-year-old woman who was previously operated on for a macular hole and was referred for reoperation. Optical coherence tomography revealed a 622 μm macular hole in her left eye. Following discussions on the risk, intentional retinal detachment and posterior capsule transplantation were performed. Postoperative optical coherence tomography confirmed the closure of the macular hole and improvement in symptoms. The combination of intentional retinal detachment and posterior capsule transplantation is helpful and a novel technique for refractory macular holes that do not close after initial surgery.

## Introduction

Full-thickness macular holes (FTMH) are foveal defects involving all retinal layers. Their formation is primarily attributed to incomplete posterior vitreous detachment (PVD), which results in persistent anteroposterior and tangential vitreofoveal traction. This traction causes foveal dehiscence, leading to the development of a FTMH.

Key risk factors for idiopathic macular hole formation include advanced age, female sex, high myopia, trauma, and a history of macular hole in the fellow eye. Macular holes are classified based on Gass's staging system, ranging from Stage 1 (impending hole) to Stage 4 (full-thickness hole with complete PVD) [1].

Internal limiting membrane (ILM) peeling has become the standard technique in vitrectomy for macular holes, with a reported closure rate of 94% [2]. Furthermore, the Inverted ILM flap technique has become the preferred approach for large macular holes with a diameter of 400 μm or greater, achieving a reported hole closure rate of 98% [3]. However, the inverted ILM flap technique cannot be performed on unclosed macular holes already undergoing ILM peeling.

For these refractory macular holes, there are reports of using a coating material, such as free ILM implanted into the hole [4], lens capsule implants [5], autologous retinal allografts [6], or human amniotic membrane [7].

Previous studies have shown that even in the era of small-gauge vitrectomy with ILM peeling, macular hole recurrence occurs in approximately 3.3% of cases. Risk factors include high myopia, cataract surgery, and cystoid macular edema. Furthermore, epiretinal membrane (ERM) formation and bilateral involvement are also commonly observed in these patients [8].

Moreover, non-closed macular holes after vitrectomy are thought to form secondary firm adhesions between the contracted retina and the retinal pigment epithelium (RPE). For such adhesions, there are reports of intentionally creating retinal detachment by injecting subretinal fluid to release the adhesion between the retina and the RPE, thereby facilitating closure of the macular hole [9].

Here, we present two unique cases of refractory macular holes in highly myopic eyes that were successfully treated using a novel combination of intentional retinal detachment and posterior lens capsule transplantation. To our knowledge, this is the first report to combine these two techniques, offering a new strategy for cases where conventional approaches are no longer applicable.

## Case presentation

Case 1

A 60-year-old man presented with the chief complaint of decreased visual acuity in both eyes. His medical history revealed that he became aware of vision loss three years prior and consulted a local doctor. He was diagnosed with a macular hole in both eyes and was referred to our clinic.

**Figure 1:**
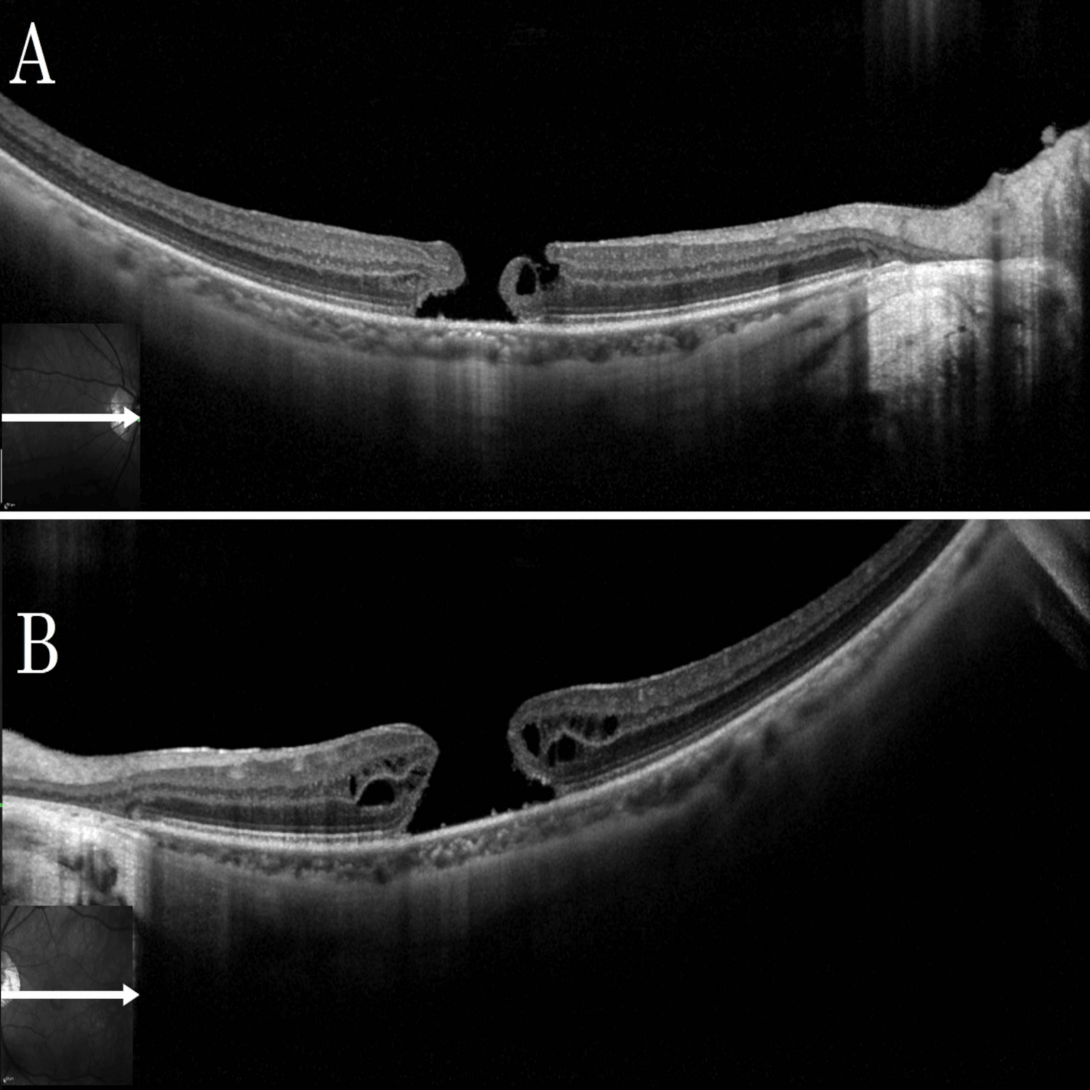
Baseline OCT Images of the Right and Left Eyes Showing Large Macular Holes in Case 1 Case 1: (A) Optical coherence tomography (OCT) of the right eye at the initial visit showed a macular hole with a basal diameter of 962 μm. (B) OCT of the left eye at the initial visit showed a large macular hole with a basal diameter of 1451 μm.

Initial findings included a best-corrected visual acuity (BCVA) of 0.52 logMAR for the right eye and 0.70 logMAR for the left eye. Refraction of the right eye was −9.00 D sphere, −0.50 D cylinder at 80°, and the left eye −8.50 D sphere, −0.75 D cylinder at 80°. The intraocular pressure was 14 mmHg in the right eye and 17 mmHg in the left eye, and the axial length was 28.4 mm in the right eye and 27.9 mm in the left eye, indicating high myopic eyes. Emery-Little classification Grade II nuclear sclerosing cataracts were observed in both eyes. Color fundus photographs at the time of initial examination showed macular holes in both eyes, and optical coherence tomography (OCT) revealed large macular holes with a basal diameter of 962 μm in the right eye (Figure 1A) and 1451 μm in the left eye (Figure 1B).

The clinical course involved a 27-gauge (G) vitrectomy with concomitant cataract surgery performed on the right eye, which had a smaller diameter macular hole. The right eye underwent ILM peeling around the macular hole, except for the upper area of the fovea. Then, the ILM above the macular hole was inverted to cover the macular hole. A fluid-air exchange was performed, and the patient was instructed to remain in the prone position for two days postoperatively.

**Figure 2:**
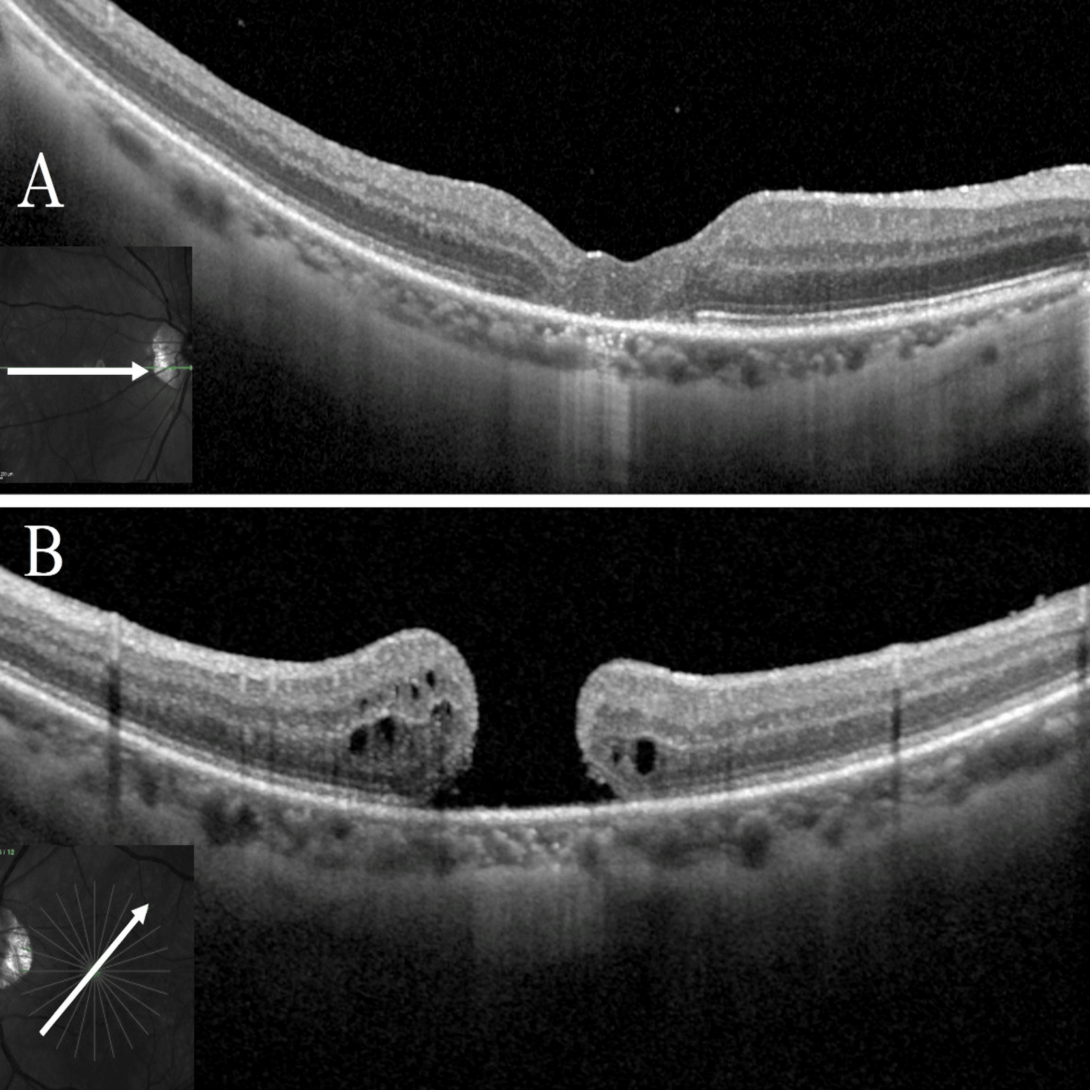
Postoperative OCT Findings of Both Eyes on Day 27 in Case 1 Case 1: (A) Optical coherence tomography (OCT) of the right eye on postoperative day 27 confirmed the closure of the macular hole. (B) OCT of the left eye on postoperative day 27 showed an unclosed macular hole with a basal diameter of 986 μm.

OCT examination on postoperative day 27 confirmed the closure of the macular hole (Figure 2A). The left eye underwent a similar 27-G vitrectomy with concomitant cataract surgery one week after the right eye surgery. After ILM peeling around the macular hole, epiretinal proliferation (EP) was observed at the margin and was intentionally inserted into the macular hole to promote closure. Gas tamponade was then performed using 20% sulfur hexafluoride. The patient was asked again to stay prone for nine days after the surgery. However, at an outpatient visit on the 27th postoperative day, an unclosed macular hole with a basal diameter of 986 μm remained in the left eye (Figure 2B).

The patient underwent revision surgery with a 27-G vitrectomy (Video 1).

**Video 1:** Posterior Capsule Transplantation Technique in Case 1 The surgery begins with the creation of an intentional retinal detachment at the temporal and inferior sides of the macular hole. The posterior lens capsule, stained with Brilliant Blue G, is then carefully removed using vitreous forceps and placed directly over the macular hole to cover it. Subsequently, viscoelastic material is applied over the capsule, followed by fluid-air exchange and SF6 gas tamponade.

**Figure 3:**
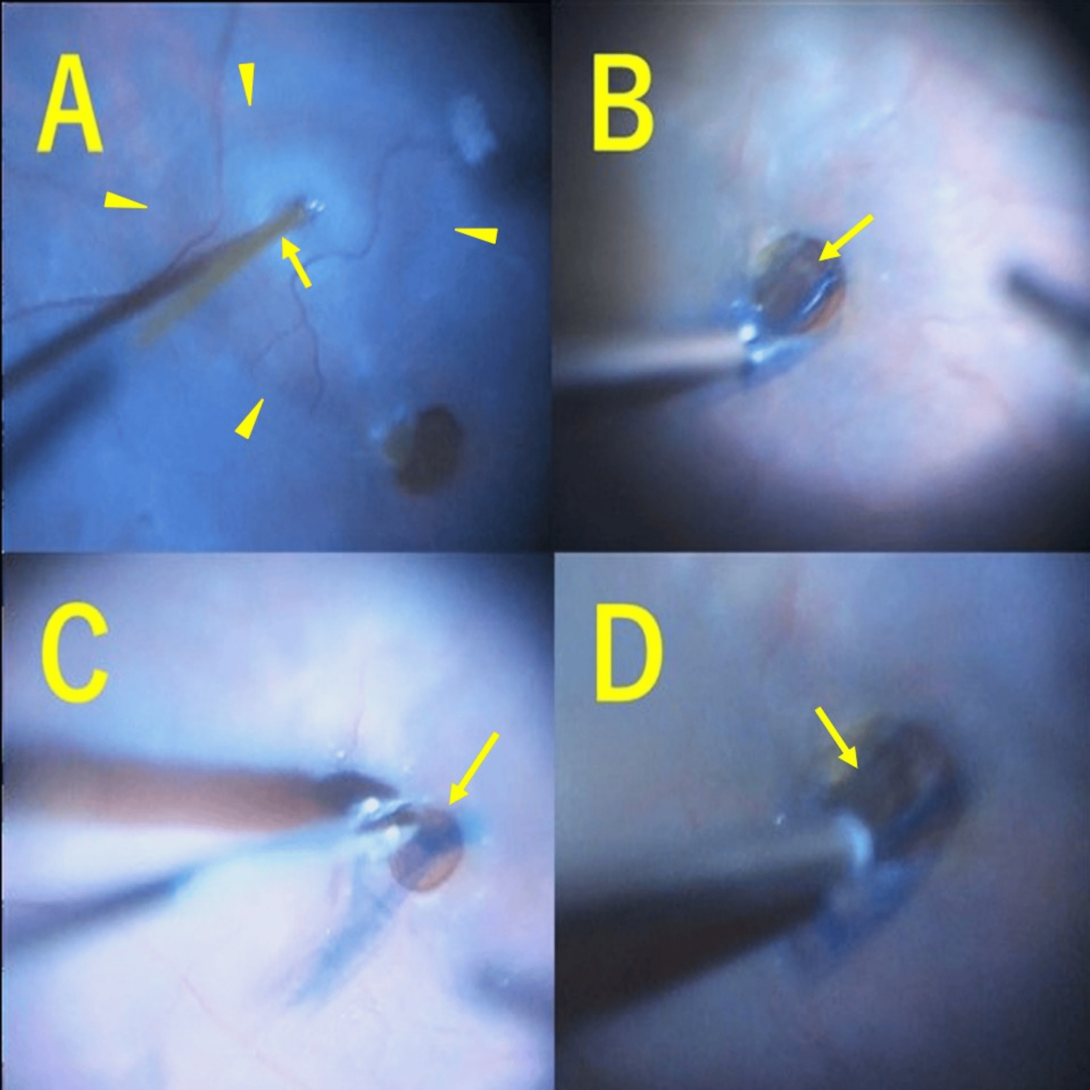
Key Intraoperative Steps for Posterior Capsule Transplantation in the Left Eye (Case 1) (A) An intentional retinal detachment (arrowhead) was created using a 38-gauge subretinal injection needle (arrow) centered temporally and inferiorly to the macular hole in the left eye. (B) The posterior lens capsule stained with Brilliant Blue G (arrow) was fixed over the macular hole. (C) Part of the posterior capsule was tucked under the retina adjacent to the macular hole (arrow). (D) A viscoelastic material was added over the posterior capsule to stabilize it (arrow).

**Figure 4:**
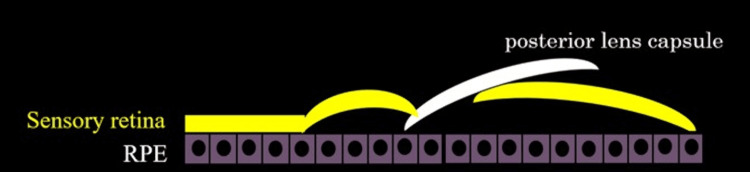
Schematic of Posterior Capsule Insertion for Macular Hole Closure Schematic illustration showing the insertion of the posterior capsule between the lower retinal edge of the macular hole and the RPE after intentional retinal detachment. This technique enables the precise placement of the capsule over the macular hole, thereby preventing displacement. RPE: retinal pigment epithelium. Image created by the authors.

In the reoperation on the left eye, balanced salt solution (BSS PLUS®, Alcon Laboratories, Fort Worth, TX, USA) was injected under the retina using a 38-gauge subretinal injection needle (Surgical Polytip Cannula; MedOne Surgical, Sarasota, FL, USA), centered temporally and inferiorly to the macular hole, to create an intentional retinal detachment (Figure 3A) and to release the adhesion between the EP and the RPE at the edge of the macular hole. A new ILM detachment was attempted but abandoned. Next, a viscoelastic material (purified sodium hyaluronate chondroitin sulfate) was implanted over the macular hole. At the same time, the posterior lens capsule, stained with Brilliant Blue G, was excised using vitreous forceps and directly carried onto the macular hole to cover it (Figure 3B). A portion of the posterior capsule was tucked under the nasal retina (Figure 3C). A viscoelastic material was then placed on the posterior capsule to stabilize it (Figure 3D), and the position of the posterior capsule was adjusted and fixed (Figure 4).

**Figure 5:**
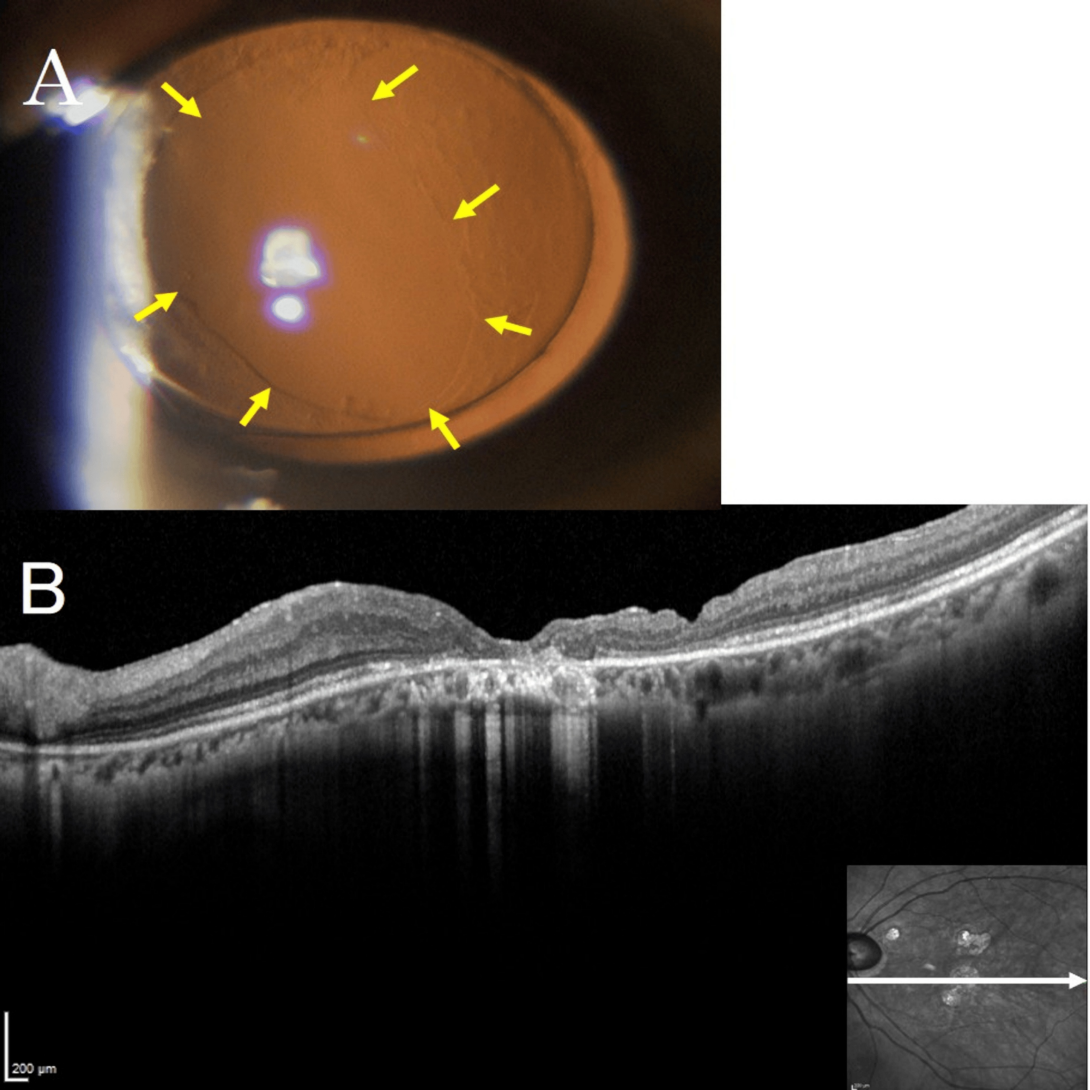
Slit Lamp and OCT Findings After Surgery in Case 1 (A) A slit lamp examination showed the removed posterior lens capsule. The yellow arrow indicates the edge of the excised posterior lens capsule. (B) Closure of the macular hole was observed by optical coherence tomography. OCT: optical coherence tomography.

After fluid-air exchange, a 9% octafluoropropane gas tamponade was conducted, and the patient was placed in the right lateral supine position for several hours postoperatively, followed by a prone position for one week postoperatively. Macular hole closure was observed by swept-source OCT on the day after reoperation. Postoperative OCT confirmed successful closure of the macular hole. Two months after reoperation, BCVA in the left eye improved to 0.30 logMAR. Refraction was −2.00 D sphere, −0.75 D cylinder at 10°. A slit lamp examination showed the removed posterior capsule (Figure 5A), and the closure of the macular hole was observed by OCT (Figure 5B).

Case 2

A 69-year-old woman presented with a chief complaint of metamorphopsia (visual distortion) in the left eye. In her medical history, she had undergone a vitrectomy for a macular hole in the left eye at another hospital three years earlier, but the hole had not closed, and there was no improvement in vision in that eye.

**Figure 6:**
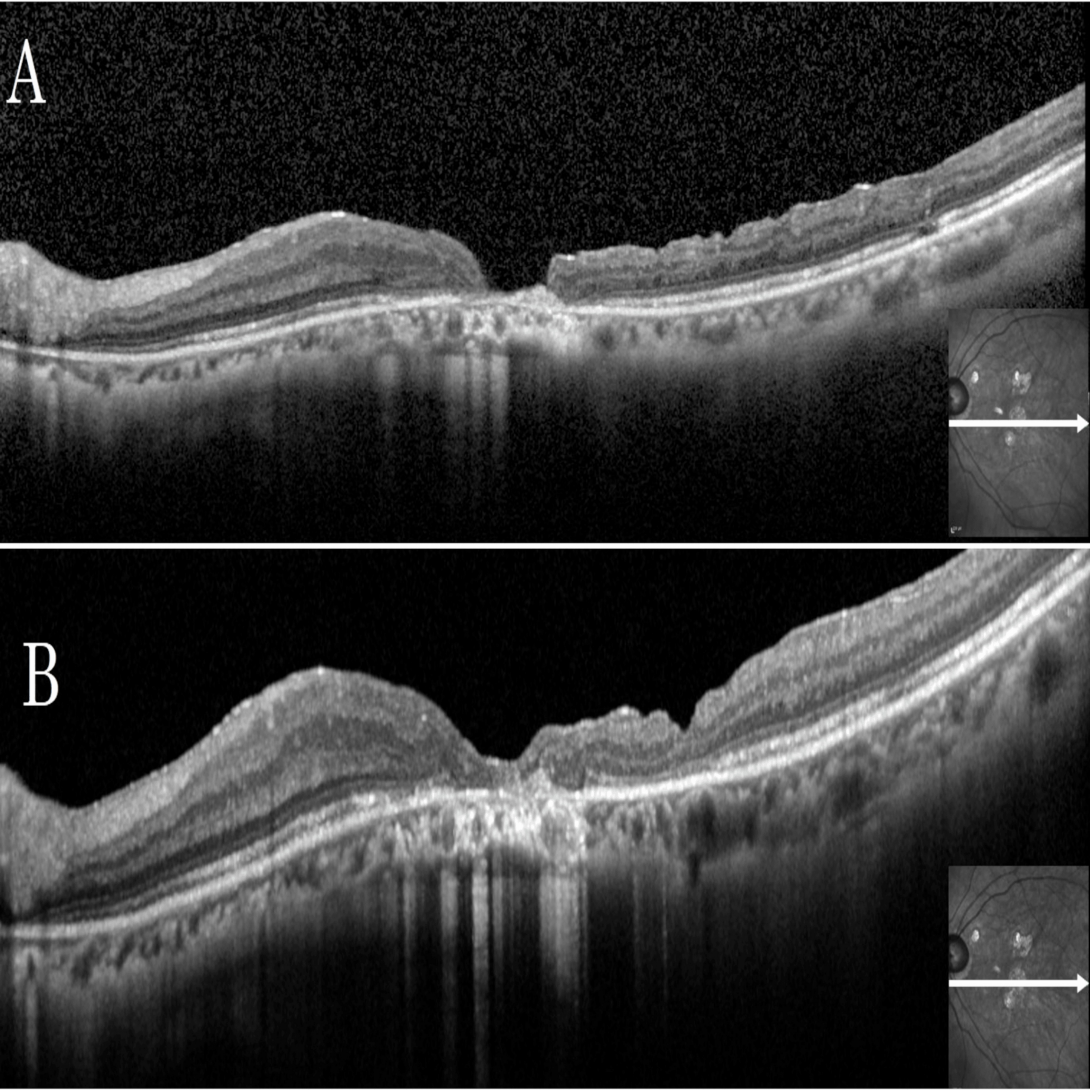
Pre- and Postoperative OCT Images of the Left Eye in Case 2 (A) Optical coherence tomography (OCT) of the left eye at the initial visit showed a macular hole with a basal diameter of 622 μm. (B) OCT three months postoperatively showed closure of the macular hole.

Initial BCVA was 0.16 logMAR in the right eye and 0.70 logMAR in the left eye. Refraction of the right eye was -2.25 D cylinder at 170°, and the left eye was +2.50 D sphere/-2.00 D cylinder at 65°. Intraocular pressure was 16 mmHg in both eyes, axial length was 28.0 mm in the right eye and 27.5 mm in the left eye, both eyes had intraocular lenses inserted, and posterior capsular opacity (PCO) was observed in the left eye. A color fundus photograph of the left eye showed a macular hole and scattered retinal pigment epithelial atrophy around the hole, and OCT showed a Stage 4 macular hole with a hole base diameter of 622 μm and flattened hole margin in the left eye (Figure 6A).

The clinical course involved explaining the potential risk of paracentral visual field defect enlargement associated with intentional retinal detachment during reoperation, and considering the patient's strong desire for treatment, a 27-G vitrectomy was performed (Video 2).

**Video 2:** Posterior Capsule Transplantation Technique in Case 2 The same surgical procedure as in Case 1 was performed, including intentional retinal detachment, posterior capsule placement, viscoelastic application, and gas tamponade.

Intraoperatively, no obvious ERM or EP was identified. Although ILM staining was performed with Brilliant Blue G in an attempt to identify any remaining ILM edge for possible ILM transplantation, no identifiable ILM edge could be found, likely due to both prior ILM peeling in the initial surgery and severe retinal thinning. Therefore, ILM transplantation was not possible. Using the same method as in Case 1, after creating an intentional retinal detachment, the resected posterior capsule was fixed to the bottom of the macular hole, and tamponade was performed with 9% octafluoropropane. The patient was placed in a strict supine position for three days postoperatively, followed by lateral positioning on either side. Closure of the macular hole was observed the day after surgery.

**Figure 7:**
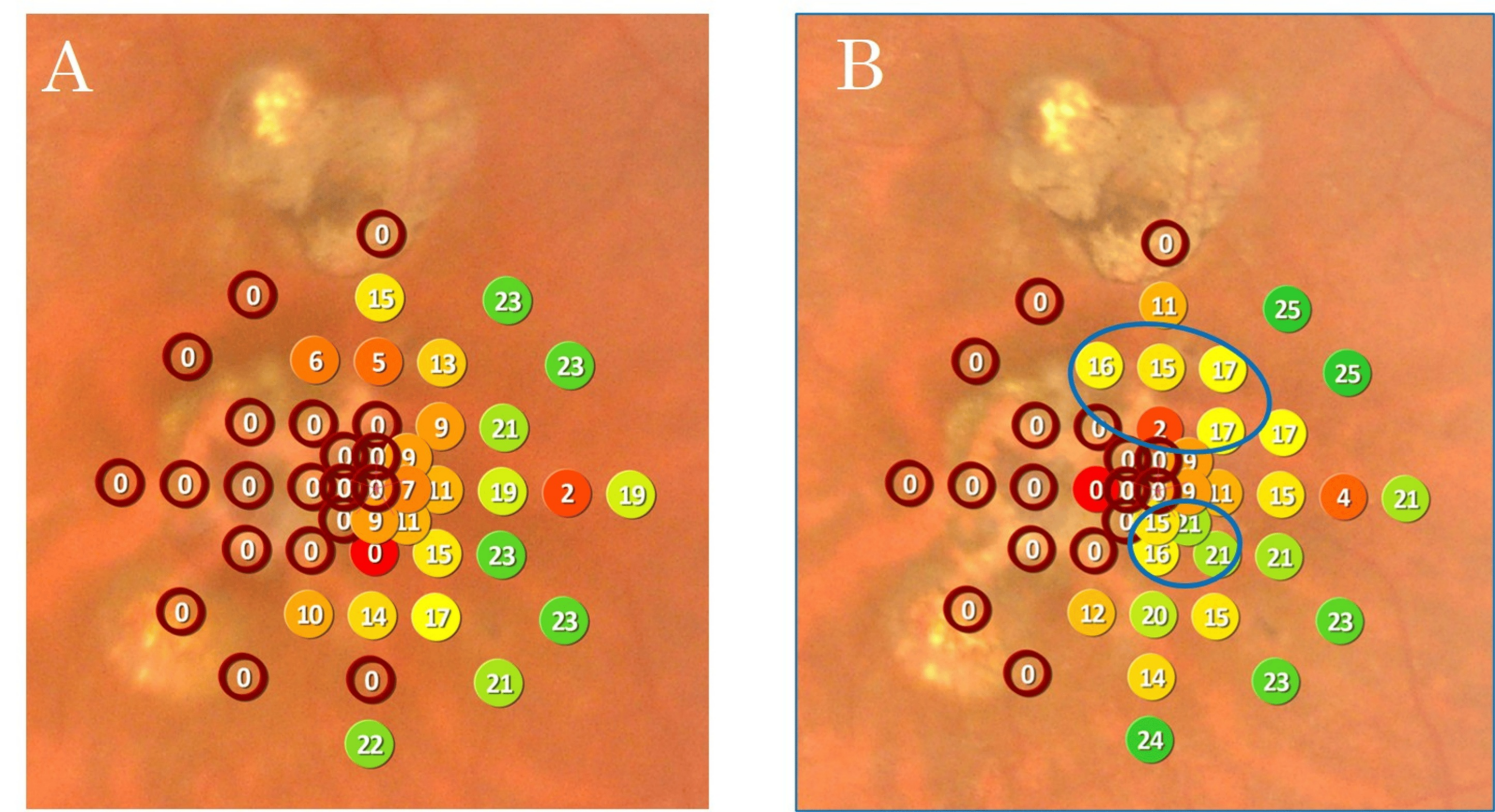
Comparison of Preoperative and Three-Month Postoperative Microperimetry (A) Preoperative MP-3 map showing decreased retinal sensitivity surrounding the macular hole. (B) Three-month postoperative MP-3 map showing increased sensitivity above and below the central fovea. The blue circles indicate areas with improved retinal sensitivity.

OCT at three months postoperatively also confirmed macular hole closure (Figure 6B). Although the left eye BCVA remained unchanged at 0.70 logMAR, the refraction was -0.50 D sphere/-1.00 D cylinder at 170°, and the patient reported improvement in metamorphopsia. A comparison of pre- and three-month postoperative microperimetry showed increased sensitivity above and below the central fovea, consistent with the subjective improvement (Figure 7).

## Discussion

Recurrent or non-closed macular holes remain a surgical challenge, even in the era of small-gauge vitrectomy with ILM peeling. In such refractory cases, alternative surgical strategies are required to achieve anatomical closure and functional recovery.

When standard techniques like the inverted ILM flap are not feasible, such as in eyes where the ILM has already been removed, several graft materials have been explored, including free ILM transplantation [4], lens capsule [5], autologous retinal grafts [6], or human amniotic membrane [7]. Among these, free ILM and lens capsules are relatively easy to harvest and manipulate intraoperatively. Furthermore, lens capsules have been reported to provide greater visual improvement than free ILM grafts [10]. This is thought to be partly due to the advantage the lens capsule has with a heavier specific gravity than the ILM, and it is easier to settle within the macular hole [5,10].

In addition to mechanical stability, the biological properties of the implanted material may also play a role in the closure of macular holes. Basement membrane components, such as fibronectin, type IV collagen, and laminin, expressed in the ILM are involved in the proliferation and migration of Müller cells during macular hole closure [11]. On the other hand, fibronectin produced by the lens epithelium induces long-term cell proliferation and fibrotic reaction, resulting in lens capsule opacity [12]. Therefore, the lens capsule may also contribute to macular hole closure, in addition to the ILM.

The creation of intentional retinal detachment has also been reported as a treatment strategy for refractory macular holes. Creating an intentional retinal detachment by injecting balanced salt solution under the subretinal space around the hole can release the adhesion between the sensory retina and the RPE, allowing the closure of the macular hole [9]. Furthermore, when measuring retinal migration distance after vitrectomy with ILM peeling and gas tamponade for a macular hole, the temporal retina migrates more than the nasal retina [13]. Therefore, we hypothesized that intentional retinal detachment on the temporal side of the macular hole would be more effective for closing the macular hole.

Based on the above, we created an intentional retinal detachment from the temporal side downward to release the adhesion between the sensory retina and RPE for the refractory macular hole that was not closed at the initial surgery. Because an ILM detachment had been performed during the initial surgery, no usable ILM was present in the vascular arcade, so the posterior lens capsule was harvested and used. The posterior capsule was inserted between the edge of the lower retina of the macular hole and the RPE after creating an intentional retinal detachment. This method ensures that the posterior capsule is placed precisely over the macular hole, preventing it from floating away. Careful follow-up is required to monitor for and minimize the risk of secondary ERM formation from the transplanted lens capsule [14]. This procedure has been conducted in only two cases, and the accumulation of more cases is needed.

## Conclusions

This report presents two cases in which intentional retinal detachment combined with posterior lens capsule transplantation was used to treat refractory macular holes. This approach may offer a feasible alternative when conventional techniques, such as the inverted ILM flap, are not applicable. By providing a new therapeutic option, this method has the potential to achieve anatomical closure and improve visual function in challenging cases. Further studies with larger patient cohorts are necessary to validate these findings and establish the broader clinical relevance of this technique.
